# Prospective study and validation of early warning marker discovery based on integrating multi-omics analysis in severe burn patients with sepsis

**DOI:** 10.1093/burnst/tkac050

**Published:** 2023-01-15

**Authors:** Jiamin Huang, Yi Chen, Zaiwen Guo, Yanzhen Yu, Yi Zhang, Pingsong Li, Lei Shi, Guozhong Lv, Bingwei Sun

**Affiliations:** Department of Burns and Plastic Surgery, Affiliated Suzhou Hospital of Nanjing Medical University, Suzhou 215002, Jiangsu Province, China; Department of Burns and Plastic Surgery, Affiliated Huaian No.1 People’s Hospital of Nanjing Medical University, Huaian 223300, Jiangsu, China; Department of Burns and Plastic Surgery, Affiliated Suzhou Hospital of Nanjing Medical University, Suzhou 215002, Jiangsu Province, China; Department of Burns and Plastic Surgery, Affiliated Suzhou Hospital of Nanjing Medical University, Suzhou 215002, Jiangsu Province, China; Department of Burns and Plastic Surgery, Affiliated Hospital of Nantong University, Nantong 226000, Jiangsu, China; Department of Burns and Plastic Surgery, Northern Jiangsu People’s Hospital, Yangzhou 225001, Jiangsu, China; Department of Burns and Plastic Surgery, Affiliated Hospital of Jiangsu University, Zhenjiang 212001, Jiangsu, China; Department of Burns and Plastic Surgery, Affiliated Hospital of Jiangnan University, Wuxi 214041, Jiangsu, China; Department of Burns and Plastic Surgery, Affiliated Suzhou Hospital of Nanjing Medical University, Suzhou 215002, Jiangsu Province, China

**Keywords:** Early warning, Marker, Integrating multi-omics analysis, Severe burn, Sepsis

## Abstract

**Background:**

Early detection, timely diagnosis and rapid response are essential for case management and precautions of burn-associated sepsis. However, studies on indicators for early warning and intervention have rarely been conducted. This study was performed to better understand the pathophysiological changes and targets for prevention of severe burn injuries.

**Methods:**

We conducted a multi-center, prospective multi-omics study, including genomics, microRNAomics, proteomics and single-cell transcriptomics, in 60 patients with severe burn injuries. A mouse model of severe burn injuries was also constructed to verify the early warning ability and therapeutic effects of potential markers.

**Results:**

Through genomic analysis, we identified seven important susceptibility genes (DNAH11, LAMA2, ABCA2, ZFAND4, CEP290, MUC20 and ENTPD1) in patients with severe burn injuries complicated with sepsis. Through plasma miRNAomics studies, we identified four miRNAs (hsa-miR-16-5p, hsa-miR-185-5p, hsa-miR-451a and hsa-miR-423-5p) that may serve as early warning markers of burn-associated sepsis. A proteomic study indicated the changes in abundance of major proteins at different time points after severe burn injury and revealed the candidate early warning markers S100A8 and SERPINA10. In addition, the proteomic analysis indicated that neutrophils play an important role in the pathogenesis of severe burn injuries, as also supported by findings from single-cell transcriptome sequencing of neutrophils. Through further studies on severely burned mice, we determined that S100A8 is also a potential early therapeutic target for severe burn injuries, beyond being an early warning indicator.

**Conclusions:**

Our multi-omics study identified seven susceptibility genes, four miRNAs and two proteins as early warning markers for severe burn-associated sepsis. In severe burn-associated sepsis, the protein S100A8 has both warning and therapeutic effects.

Highlights

- Multi-omics analyses reveal warning markers for severe burn-associated sepsis.
- Neutrophils play an important role in the early stages of severe burn injuries.
- S100A8 is an early warning indicator and therapeutic target for severe burn-associated sepsis.

## Background

Burn injuries have become a major global health problem, with more than 120,000 burn-related deaths each year [1]. Burn injuries, particularly those that are severe, lead to extravasation of large amounts of bodily fluids, poor tissue perfusion, ischemia and immune dysfunction [2,3]. Sepsis caused by local or systemic infection is a secondary injury that may occur after burn injury and further aggravate the patient’s condition [4,5].

Sepsis refers to a systemic inflammatory response caused by infection [6] and is also a serious complication of shock, burns, trauma, infection or major surgery [7]. Development of sepsis can lead to septic shock and multiple organ dysfunction [8]. Patients with burn injuries are unique in that they lose their first line of defense against foreign pathogens: the skin. Consequently, burn-associated sepsis tends to be aggressive, rapid and lethal [9]. The occurrence of burn-associated sepsis poses great difficulties in clinical treatment, particularly after septic shock. Although clinicians replenish patients’ blood volume, patients nonetheless experience adverse symptoms such as hypotension, excessive lactate and oliguria [10]. Therefore, rapid recognition and treatment of burn-associated sepsis in the earliest possible stage are essential. Neutrophils are the first immune cells that respond to burn injuries and often enter the peripheral blood within several hours [11]. Owing to their rapid response to burn injuries, these cells may be critical in the early warning and treatment of burn sepsis.

Excellent early warning indicators of burn-associated sepsis are lacking. The prediction of burn-associated sepsis is performed mainly on the basis of indicators such as bacterial endotoxin, procalcitonin, C-reactive protein and arterial blood lactate level [12–15], which reflect only the current inflammatory state of the body but do not clearly indicate whether sepsis will develop in patients with burns in the longer term. The occurrence and development of burn-associated sepsis is a complex pathological process. Most published studies have been conducted at the single-omics level. However, simultaneous multi-omics analysis would more comprehensively reveal the pathological mechanisms of burn-associated sepsis development and reveal potential targets for early warning and treatment, and thus, should be valuable in guiding clinical treatment.

Here, we performed a multi-center, prospective multi-omics study in which 77 peripheral blood samples were collected from healthy and severely burned patients, and these patients were subsequently followed for sepsis. We performed comprehensive omics analyses of these samples, including genomics, microRNAomics and proteomics. Because of the important role of neutrophils in burn injuries, we performed single-cell transcriptome sequencing (scRNA-seq) on neutrophils from the peripheral blood of healthy individuals and patients with severe burn injuries. Our dataset provides a rich resource for exploring the regulatory relationships among genetic variation, transcription and translation. In addition, we identified S100A8 as a key target for early warning and treatment of burn-associated sepsis on the basis of proteomics and neutrophil single-cell transcriptomics.

## Methods

### Ethical approval

Institutional ethics committee approval for this study was received from Nanjing Medical University Affiliated Suzhou Hospital. All patients or guardians of patients and healthy volunteers signed informed consent forms prior to the collection of human blood samples. Experimental procedures were followed according to guidelines.

### Prospective design

This study is a multi-center prospective study. Based on preliminary clinical trials and statistical analysis, blood samples from severe burn patients in the burn units of five third-class hospitals in China were collected and tested. The patients were >18 years of age and admitted to the burn unit within 24 h. Acute fluid resuscitation was performed 24 h after admission. The criteria for severe burn patients were: total burn area ≥30% or third-degree burn area ≥10% or with moderate to severe inhalation injury. According to the diagnostic principle of sepsis 3.0, severe burn patients were divided into two groups, i.e. burn patients without and with sepsis.

### Human sample collection

Peripheral blood was taken from healthy donors and burn patients. Blood samples from each healthy donor was collected into EDTA anticoagulant tubes. Frozen whole blood from 40 severely burned patients was used for whole exome sequencing (WES). Peripheral blood plasma was collected from severe burn patients and healthy controls for miRNA (micro-Ribonucleic acid), data-independent acquisition (DIA) sequencing and parallel reaction monitoring (PRM) detection. All serum samples were separated by centrifugation for 10 min at 1000 g and then stored in aliquots at −80°C. Single-cell transcriptome sequencing was performed on peripheral blood neutrophils from five severe burn patients (on Burn-Day1, 2, 3) and five healthy controls.

### WES and analysis

#### Evaluation of DNA quality

A certain amount of dirty data can be found in raw sequence data. To ensure the accuracy and reliability of subsequent information analysis results, we use FASTP quality control software to quality control the original data and obtain effective clean data.

#### Detection and analysis of variation results

Starting from clean data for each sample after quality control, data were compared to a reference genome (HG19) for sequencing depth and coverage statistics. Based on the comparison results, clean data was subjected to variant detection, mainly using single nucleotide variants (SNV) and insertion and deletion (INDEL) detection. Categories 3–5 and 4–5 types of gene variation were screened for further analysis. Category 3–5 refers to screening for categories 3 (uncertain significance), 4 (likely pathogenic) and 5 (pathogenic) variations to make mutation statistics for each sample, while category 4–5 refers to screening for categories 4 and 5 variations and doing a mutation count per sample.

### Sequencing and analysis of miRNA

#### Differential expression analysis of miRNA

The data source of the cluster heatmap is the probe data of all differences in each group. The screening criterion for differential miRNAs is |fold change| > 2.

#### Gene ontology functional enrichment analysis

The top 10 miRNAs with the largest multiple of difference were selected from each group, and target gene prediction of micrornas was carried out in Targetscan, microRNA.ORG and miRDB databases respectively. Genes predicted in all three databases were selected for subsequent enrichment analysis. The *p*-values of enrichment analysis are calculated according to the Fisher exact test.

### DIA sample preparation and data analysis

#### Sample preparation

An appropriate amount of SDT buffer was added to all samples, which were then bathed in boiling water for 15 min, centrifuged at 14,000 g for 15 min, and the supernatant was filtered by 0.22 μm centrifuge. The bicinchoninic acid (BCA) method was used for protein quantification.

#### Sample quality detection

All samples were analyzed by sodium dodecyl sulfate polyAcrylamide gel electrophoresis (SDS-PAGE) electrophoresis and liquid chromatography-mass spectrometry/mass spectrometry (LC–MS/MS). A total of 20 μg of protein from each sample was added into 6× sample loading buffer and placed in boiling water for 5 min. Then, 12% SDS-PAGE electrophoresis (250 V, 40 min) and Coomassie brilliant blue staining were performed. LC–MS/MS quality inspection was performed to separate the enzymatically hydrolyzed peptides through a chromatographic column connected in series to a mass spectrometer for primary and secondary mass spectrometry analysis.

####  Data dependent acquisition (DDA) library construction

The peptides were re-dissolved in solvent A (0.1% formic acid in water) and analyzed with on-line nanospray LC–MS/MS on an Orbitrap Exploris 480 coupled to an EASY-nLC system (Thermo Fisher Scientific, MA, USA). Subsequently, 1 g of peptide sample was loaded on an analytical Nano Technology C18 analytical column (18 cm with 1.9 µm C18 Resin) (Nanjing Jinying Biotechnology Co., Catalog Number: 26350-3) and separated for 120 min with a gradient from 2 to 35% solvent B (0.1% formic acid and 80% acn). The flow rate was maintained at 300 nl/min. An electrospray voltage of 2056 V vs. the inlet of the mass spectrometer was used.

#### Principal component analysis

Principal component analysis (PCA) is a statistical analysis method that is usually used to reflect intra-group and inter-group differences in samples. The higher the degree of aggregation of samples within the same group the better the parallelism or repeatability of samples within the group. Differences in the relative positions of samples from different groups indicate significant group differences between the different groups.

#### Differential expression analysis of protein

In the analysis of quantitative results, data with at least 25% non-null values were screened for statistical analysis. Quantile standardization is carried out for sample data, log_2_ is taken for quantitative values and null values are filled with minimum values. Statistical methods of linear model fitting and Bayesian tests were used to perform variance analysis. Proteins that met the screening criteria of expression differential multiple >1.2 times (up − down) and *p* value < 0.05 were defined as differential proteins.Gene ontology and Reactome pathway annotation

The software g:Profiler2 was used to perform functional enrichment analysis of differential proteins. The distribution and significance of target protein sets in each gene ontology (GO) classification or Reactome pathway were analyzed by Fisher’s exact test.

#### Cluster analysis

Firstly, the quantitative information of the target protein set was normalized before cluster analysis. Secondly, pheatmap software was used to classify both samples and protein expression levels. Finally, the hierarchical clustering heatmap was generated.

#### PRM protein detection

For each sample, Dithiothreitol (DTT) was added to ~150 μg of protein, to a final concentration of 100 mM. Samples were boiled for 5 min and cooled to room temperature. Subsequently, each sample was mixed with 200 μl of UA buffer (8 M urea [Bio-Rad, 161–0731] and 150 mM Tris–HCl, pH 8.0) and centrifuged in an ultrafiltration tube at 12,500 × g for 15 min. The filtrate was discarded and 100 μl of Iodoacetamide (IAA) buffer was added (100 mM IAA [Bio-Rad, 163-2109] in UA) and shaken for 1 min at 600 rpm. Each sample was incubated at room temperature for 30 min and centrifuged at 12,500 × g for 15 min. Each sample was washed with 100 μl of UA buffer and 100 μl of NH_4_CO_3_ (40 mM) buffer twice at 12,500 × g for 15 min. Subsequently, 40 μl of trypsin-containing NH_4_CO_3_ buffer was added (1:50; Promega, 317,107), shaken at 600 rpm for 1 min and incubated at 37°C for 16 h. After centrifugation at 12,500 g for 15 min, 40 μl of NH_4_HCO_3_ buffer was added and incubated for 15 min and the peptides were collected. After desalting and lyophilization, the peptides were re-dissolved in buffer A and the peptide content was estimated on the basis of UV spectral density at 280 nm. The peptide mixture was loaded on an analytical column (Thermo Fisher Scientific, Acclaim PepMap RSLC 50 μm × 15 cm, Nano Viper, P/N164943) and separated at 300 nl/min for 2 min (from 5 to 10% buffer B), a 43 min linear gradient (from 10 to 30% buffer B) and a 10 min linear gradient (from 30 to 100% buffer B), then maintained at 100% buffer B for 5 min. Liquid chromatography-parallel reaction monitoring/mass spectrometry (LC-PRM/MS analysis) (Aptbio, Shanghai, China) was performed with a Q-Exactive HF mass spectrometer (Thermo Scientific, USA) coupled to an Easy-nLC 1000 instrument (Thermo Scientific, USA) for 60 min. The mass spectrometer used positive-ion mode. The resolution of the full MS scan was 60,000, the scan mass range was 350–1500 m/z, the target Automatic gain control (AGC) was 1e6 and the maximum injection time was 50 ms. After each full MS scan, 10 PRM scans (MS2 scans) were collected according to the inclusion list with an isolation window of 1.6 m/z, a resolution of 15,000, a target AGC of 1e5 and a maximum injection fill time of 50 ms. The peptides were selected for MS2 scanning, Higher energy collision induced dissociation (HCD) working mode was used and the normalized collision energy was set to 27%.

#### Receiver operating characteristic curve drawing

The receiver operating characteristic (ROC) curve is an important tool for diagnostic test evaluation, providing complete and easy visualization of sensitivity/specificity reports. In the ROC curve, the true positive rate (sensitivity) is plotted as a function of the false positive rate (specificity) for different cut-off points for a given parameter. Each point on the ROC curve represents a sensitivity/specificity pair corresponding to a specific decision threshold. A test with complete discrimination (no overlap in the two distributions) has a ROC curve (100% sensitivity, 100% specificity) through the upper left corner. Therefore, the closer the ROC curve is to the upper left corner the higher the overall accuracy of the test. The closer the ROC curve is to the diagonal the less diagnostic the test is. The area of the ROC curve is the degree to which a parameter differentiates two diagnostic groups (disease group/normal group).

#### Animals

A total of 150 male C57BL/6 J mice animals were obtained from the Animal Center of Nanjing Medical University, weighing between 20 and 25 g and 8–10 weeks old. A pathogen-free environment is provided for all mice. Animals have unlimited access to food and water. Animal feeding and sacrifice methods were approved by the Animal Welfare Ethical Review Committee of Nanjing Medical University.

#### Experimental models of scald burns

Each mouse back was depilated with a shaver after anesthesia with isoflurane. The back of each mouse was heated at 100°C for 8 s. A mouse burn model was successfully established with 30% body surface area scald burn inflicted. Saline (1 ml) was administered to the mice as part of their resuscitation.

#### Experimental design

In the first set of experiments, mice were divided into two groups: sham group (Sham, *n* = 5) and burn group (Burn, *n* = 20). Lung, spleen, liver and kidney of mice were collected for pathological examination at 8 h and 1 day postoperatively.

In the second experiment, 44 mice were randomly divided into three groups: sham group (Sham, *n* = 12), burn group (Burn, *n* = 16) and burn+ paquinimod (PAQ) group (Burn+PAQ, *n* = 16). One hour before the establishment of the burn model, the mice in the Burn+PAQ group were injected with 10 mg/kg PAQ (dilution scheme: 10% Dimethyl sulfoxide (DMSO) with PAQ + 40% PEG300 + 5% Tween-80 + 45% saline). Sham and Burn groups were injected with a diluted solution without PAQ. The mice in the three groups were injected each day and the survival rate of the mice was observed during the study period. The specific experimental procedure is presented in Figure 8a.

In the third set of experiments, mice were divided into three groups: sham group (Sham, *n* = 20), burn group (Burn, *n* = 35) and burn+PAQ group (Burn+PAQ, *n* = 35). The injection regimen of the three groups was the same as that of the first experiment. During the experiment, the dead mice are promptly removed. Blood samples and organs of mice were collected on the first, third and seventh day after operation for subsequent experiments.

#### Histopathological analysis

The mice were anesthetized with isoflurane and transcardially perfused with saline and 4% paraformaldehyde. Subsequently, various organs of the mice were fixed with 4% paraformaldehyde overnight at 4°C. Samples were routinely dehydrated, embedded in paraffin, sectioned and stained with hematoxylin–eosin (H&E). The pathological changes of each organ were observed under a light microscope.

#### Flow cytometry

White blood cells in mouse peripheral blood were collected using red blood cell lysis (Biyuntian, China). The white blood cells were suspended in precooled phosphate buffered saline (PBS) at a concentration of 2 × 10^6^ cells/ml (100 μl of PBS, 2 × 10^5^ cells/tube). Ly6G and CD177 antibodies (BD Bioscience, USA) were used according to the manufacturer’s protocol. After 30 min incubation at 4°C in the dark, cells were washed and analyzed on a FACS Canto II cytometer (BD Biosciences, USA). The data were analyzed with the FlowJo software (FlowJo).

#### Detection of S100A8 in serum of mice by enzyme-linked immunosorbent assay (ELISA)

Peripheral blood was collected by orbital blood sampling and plasma was obtained after centrifugation. The expression level of S100A8 in plasma was detected by ELISA; the kit was provided by Hangzhou Lianke Biotechnology Co. (Hangzhou, China).

#### Cytometric beads array for detecting cytokine levels

Cytometric Beads Array Mouse Th1/Th2/Th17 Cytokine Kit (BD Pharmingen) was used to detect the levels of interleukin-2 (IL-2), IL-4, IL-6, nterferon-γ (IFN-γ), tumor necrosis factor (TNF), IL-17A and IL-10 proteins in peripheral blood plasma of cultured mice according to the manufacturer’s instructions. Data were obtained in FACS CANto-II (BD Bioscience, USA). The data were analyzed with the FlowJo software (FlowJo).

### Statistical analyses

GraphPad Prism 7 was used for statistical analysis. All of the data are presented as the mean ± standard deviation. Student’s t test or Mann–Whitney U test was used to compare the differences between two groups. One-way analysis of variance tests were used for three or more group comparisons. A value of *p* < 0.05 was considered to be statistically significant.

## Results

### Comprehensive multivariate analysis of patients with severe burn injuries

**Figure 1:**
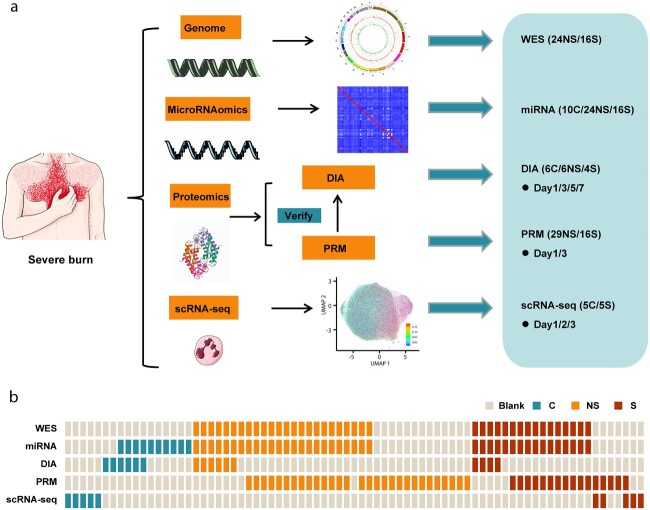
Multi-omics landscape of severe burn patients. (**a**) Overview of the experimental design. (**b**) The number of samples for genomics, microRNAomics, proteomics and scRNA-seq analyses. *WES* whole exome sequencing, *DIA* data-independent acquisition, *PRM* parallel reaction monitoring, *scRNA-seq* single-cell transcriptome sequencing, *C* control, *NS* burn group without sepsis, *S* burn group with sepsis

This project was a multi-center, prospective multi-omics study. From June 2018 to October 2020, blood samples were collected from 60 patients with severe burn injuries (inclusion criteria: total burn area ≥30% or third-degree burn area ≥10% or moderate to severe inhalation injury) and 17 healthy volunteers from five tertiary hospitals in China. On the basis of the diagnostic principles of sepsis 3.0, the patients were divided into a burn group without sepsis (NS group, *n* = 37) and a burn group with sepsis (S group, *n* = 23). Clinical data were collected during follow-up. Data were collected on the demographic characteristics (age and sex) of patients with burn injuries and the degree of the burn (burn depth and area, and presence or absence of inhalation injury). The 77 samples were subjected to comprehensive multi-omics analysis, including genomics, microRNAomics, proteomics and scRNA-seq. Genomics analysis was performed on whole blood cells from peripheral blood samples; microRNAomics and proteomics were used to analyze peripheral blood plasma samples; and scRNA-seq was performed to detect circulating neutrophils. Proteomic analysis focused on two aspects: DIA and PRM. The purpose of DIA analysis is to screen for differential proteins, whereas the purpose of PRM detection is to accurately verify the previous differential proteins. A schematic diagram of the experimental design is shown in Figure 1a and the corresponding relationships among samples are shown in Figure 1b.

### Genomic landscape of severe burn patients

**Figure 2:**
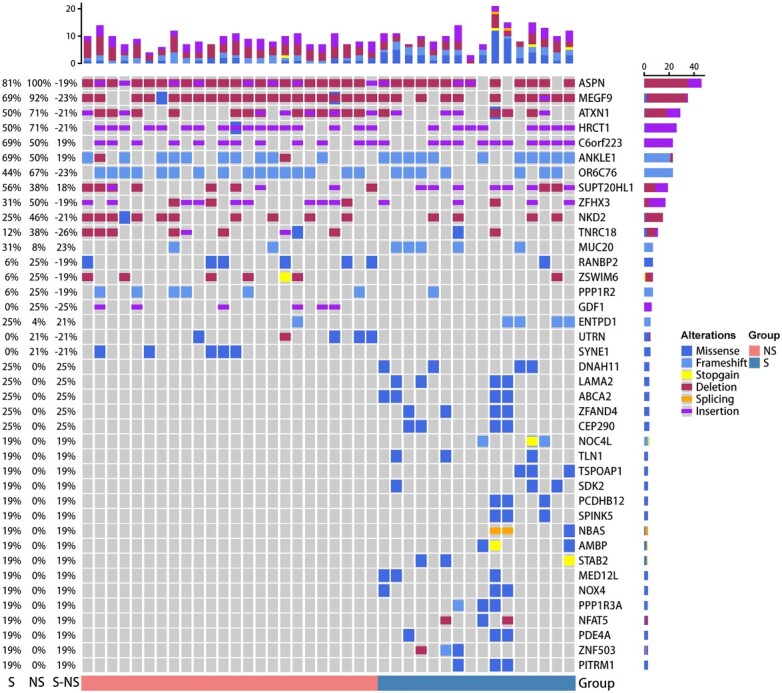
Genomic analysis of patients with severe burn injuries. Genomic profiles. Top: counts of the mutated genes in each patient; bottom: group assignment for 40 patients. Mutation frequency is shown by the bar chart in the left panel and mutation type is shown by the bar chart in the right panel. *NS* burn group without sepsis, *S* burn group with sepsis

To understand the differences in genomics between the NS and S groups, we selected peripheral blood whole blood cells from 40 severe burn patients (NS group: *n* = 24, S group; *n* = 16) for genomic analysis according to strict criteria. The details of patients with burn injuries are shown in Table 1. In this study, WES was used to detect primarily two types of variants: single nucleotide variants and insertions and deletions. According to the Guidelines of the American College of Medical Genetics and Genomics, the variant sequences were divided into five types. In this study, we focused on the 3–5 and 4–5 types of gene variants. A total of 4621 mutated genes were screened for category 3–5 variation and 122 mutated genes were screened for category 4–5 variation. We screened genes with a difference of >18% between the S group and the NS group. A total of 26 susceptibility genes were found, among which the seven susceptibility genes with the most significant differences were DNAH11, LAMA2, ABCA2, ZFAND4, CEP290, MUC20 and ENTPD1 (Figure 2). Mutations in these genes may increase the risk of sepsis in patients with severe burn injuries.

**Table 1:** Characteristics of burn patients grouped by sepsis according to WES and miRNA analysis. Data presented as median (age range); number (percentage)

**Characteristics**	**Healthy control**	**NS group**	**S group**
	*n* = 10	*n* = 24	*n* = 16
Age, years	28.5 (19–42)	53.5 (18–83)	51.5 (28–62)
%TBSA	NA	30–80	35–98
Degree of burn	NA	II–III	II–III
Sex, male (%)	1 (10)	18 (75)	11 (68.75)
Inhalation injury (yes:no)	NA	12:12	11:5

*TBSA* total body surface area, *NA* not applicable, *WES* whole exome sequencing, *NS group* burn group without sepsis, *S group* burn group with sepsis

### Transcriptional landscape of miRNAs in severe burn patients

**Figure 3:**
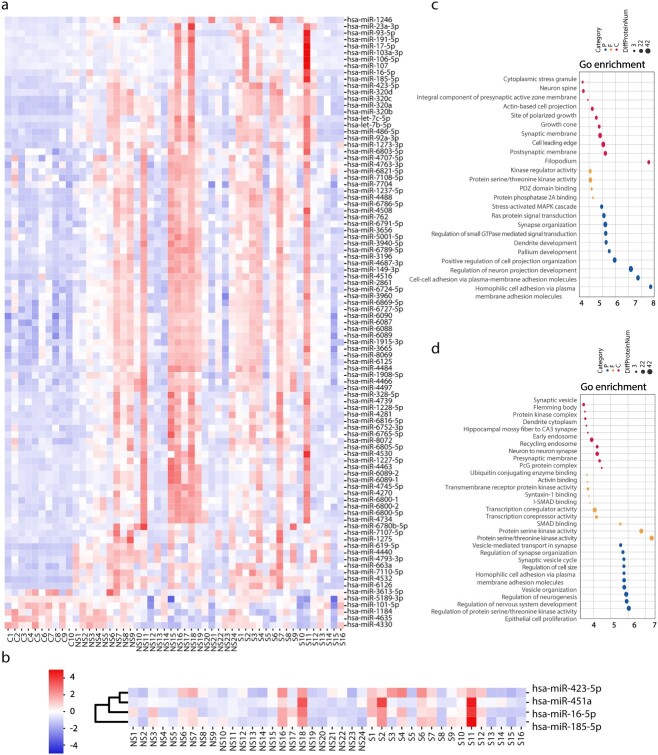
MicroRNAomics analysis of patients with severe burn injuries. (**a**) Heatmap of differential miRNAs in healthy controls and severe burn injuries. (**b**) Heatmap of differential proteins severe burn patients with sepsis or non-sepsis. (**c**) GO analysis diagram of NS/S group compared with control (C) group. The size of the circle represents the amount of protein enrichment and deeper color indicates greater functional significance. (**d**) GO analysis diagram of S group compared with NS group. The size of the circle represents the amount of protein enrichment and deeper color indicates greater functional significance. *C* control, *NS* burn group without sepsis, *S* burn group with sepsis, *GO* gene ontology

To explore the differences between severe burn patients and healthy controls, we performed miRNA detection in the peripheral blood plasma of 10 healthy controls (C group) and 40 severe burn patients (NS group: *n* = 24, S group: *n* = 16). Specific details for patients with burn injuries and healthy controls are shown in Table 1. A total of 101 differential miRNAs were screened in the NS group, as compared with group C, including 95 up-regulated miRNAs and six down-regulated miRNAs. Meanwhile, 86 up-regulated miRNAs and seven down-regulated miRNAs were screened from the S group (Figure 3a). The top 10 miRNAs with the greatest fold difference were selected from each group to predict miRNA target genes. The results of target gene prediction were used for GO analysis. Compared with those in the C group, the target genes of miRNAs in the NS group and S groups were all enriched in small GTPase, Ras protein and Mitogenactivated protein kinases (MAPK) cascade signal transduction (Figure 3c).

To search for early warning markers of burn-associated sepsis in the NS and S groups, we conducted a comparative analysis at the miRNA level between groups. Four differentially upregulated miRNAs were enriched: hsa-miR-16-5p, hsa-miR-185-5p, hsa-miR-451a and hsa-miR-423-5p (Figure 3b). GO analysis indicated that the target genes of these four miRNAs were enriched primarily in the activity of various protein kinases (threonine, serine and tyrosine kinases) and vesicle-mediated transport in synapses (Figure 3d). Previous studies have shown that threonine and serine protein kinases may potentially be associated with the occurrence and development of sepsis [16,17].

### Proteomic characteristics and commonality of severe burn patients at different time points

**Figure 4:**
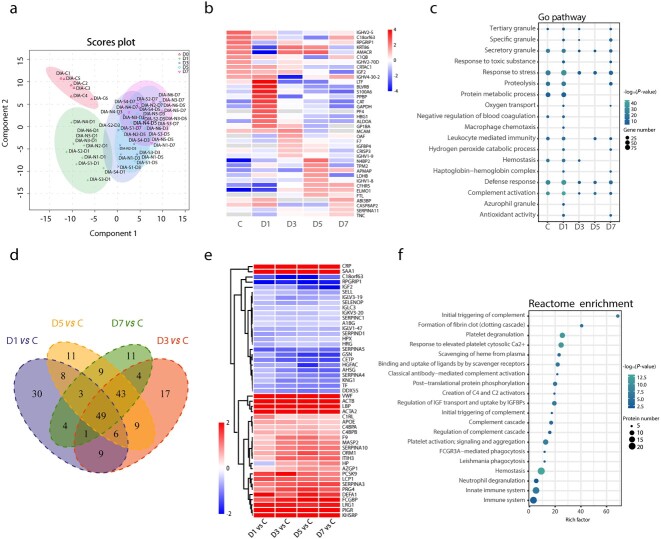
Similarities and differences of severe burn patients at different time points. (**a**) PCA score plot of severe burn patients [days (D) 0, 1, 3, 5, 7]. (**b**) Heatmap of differential proteins in healthy controls and severe burn injuries. (**c**) GO analysis diagram of different groups. The size of the circle represents the amount of protein enrichment and deeper color indicates greater functional significance. (**d**) Venn diagram of differential proteins on days (D) 1, 3, 5 and 7 of severe burn patients compared with healthy controls (group C). (**e**) Heatmap of common differential proteins in severe burn injuries compared with healthy controls. (**f**) Reactome enrichment analyses of common differential proteins. *DIA* data-independent acquisition, *C* control, *GO* gene ontology

To explore the differences and similarities in peripheral blood proteins at different time points in patients with severe burn injuries, we performed DIA detection on the peripheral blood plasma of 6 healthy controls and 10 patients at 1, 3, 5 and 7 days after extensive burn injuries. The samples were analyzed with SDS-PAGE and LC–MS/MS before DIA detection and the qualified samples were used for subsequent experiments. The details for healthy controls and patients with burn injuries are shown in Table 2. PCA revealed the main distribution characteristics of differential proteins in the peripheral blood at five time points after burn injuries (the healthy control group was considered to represent day 0 after burn injuries, Figure 4a). We observed significant differences in peripheral blood proteins between severe burn patients and healthy controls. In addition, as the disease progressed, the body transitioned from the burn shock stage to the infection stage, and the proteins secreted into the peripheral blood significantly changed (Figure 4b). The differences were most significant on the first day after severe burn injuries.

**Table 2:** Characteristics of burn patients grouped by sepsis according to DIA analysis. Data presented as median (age range); number (percentage)

**Characteristics**	**Healthy control**	**NS group (D1,3,5,7)**	**S group (D1,3,5,7)**
	*n* = 6	*n* = 6	*n* = 4
Age, years	30 (27–42)	52.5 (34–70)	50.5 (46–54)
%TBSA	NA	32–70	35–88
Degree of burn	NA	II–III	II–III
Sex, male (%)	0 (0)	6 (60)	3 (75)
Inhalation injury (yes:no)	NA	3:3	4:0

*TBSA*  total body surface area, *NA* not applicable, *DIA* data-independent acquisition, *NS group* burn group without sepsis, *S group* burn group with sepsis

The significance of functional differences on the first post-burn day was mainly reflected in the following three aspects. First, four distinct types of granules were found in neutrophils: azurophilic, secondary, tertiary and secretory vesicles. Compared with other time points (Burn-day 3, 5 and 7), the peripheral blood plasma of patients at Burn-day 1 contained more abundant grade 4 particles, thus, suggesting that the neutrophils at Burn-day 1 may play crucial roles in the occurrence and development of the disease. Second, compared with other time points (Burn-day 3, 5 and 7), the plasma protein at Burn-day 1 was more significantly enriched in functions such as complement activation and response to stress. Third, significant differences in the haptoglobin–hemoglobin complex at Burn-day 1, compared with other time points, might have predicted more severe erythrocyte damage (Figure 4c). In addition, 49 common differentially present proteins at different time points between patients and healthy controls were identified in Venn diagram analysis (Figure 4d, e). These differential proteins were enriched in functions including platelet degranulation, neutrophil degranulation, platelet activation, hemostasis and scavenging of heme from plasma (Figure 4f).

### Differences between severe burn patients with and without sepsis at different time points

**Figure 5:**
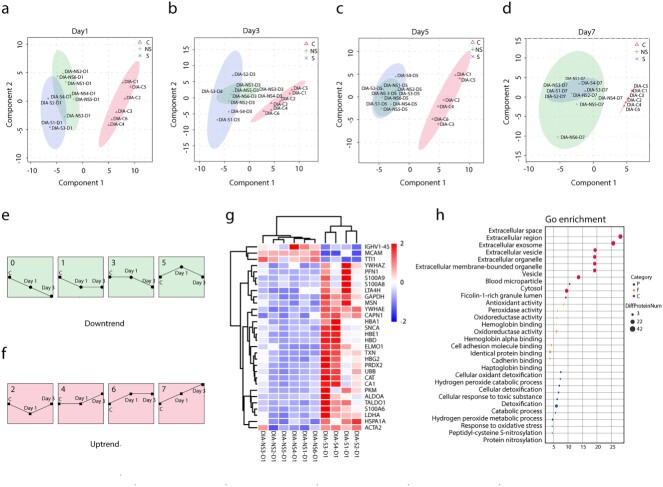
Differences between severe burn patients with and without sepsis. (**a**–**d**) PCA score plot of severe burns with sepsis and non-sepsis on days 1, 3, 5 and 7. (**e**, **f**) Trends in associated proteins at three time points (C, Day 1, Day 3) for severe burn injuries. Numbers 0, 1, 3, 5 represent uptrends, numbers 2, 4, 6, 7 represent downtrends. (**g**) Heatmap of differential proteins on day 1 of severe burn patients with sepsis and non-sepsis. (**h**) GO analysis diagram of two groups. The size of the circle represents the enrichment of the protein and the larger the value on the abscissa the greater the functional significance. *DIA* data-independent acquisition, *C* control, *NS* burn group without sepsis, *S* burn group with sepsis

To search for early warning markers of sepsis in severe burn patients, DIA sequencing was performed on the peripheral blood plasma of six healthy controls; six severe burn patients without sepsis; and four burn patients with burns without sepsis, at 1, 3, 5 and 7 days after burn injuries. PCA results indicated significant differences in major features between the S and NS groups on the first day after burn injuries (Figure 5a). On the third day after burn injuries, some differences were observed in the major characteristics between the S and NS groups (Figure 5b). However, no significant differences were observed between groups on days 5 and 7 after burn injuries (Figure 5c, d). Therefore, Burn-day 1 and Burn-day 3 were selected as the key time points to distinguish sepsis from severe burn patients.

To better identify the differences between the two groups (NS group and S group) at different time points, we conducted a trend analysis of peripheral blood plasma proteins at Burn-day 1 and Burn-day 3. Proteins conforming to the pattern 0, 1, 3, 5 were defined as downtrend proteins, and proteins conforming to the pattern 2, 4, 6, 7 were defined as uptrend proteins (Figure 5e, f). A protein with opposite trend in two groups (C-NSD1-NSD3 group and C-SD1-SD3 group) or a protein with an upward/downward trend in one group and no trend change in the other group, was defined as a trend differential protein. A total of 48 differential proteins were found by trend analysis, among which 30 proteins showed upward trends and 18 proteins showed downward trends (Table S1, see online supplementary material [18–35]). These 48 proteins were annotated and enriched in four directions (Table S2, see online supplementary material). Compared with the C-NSD1-NSD3 group, eight immunoglobulins in the C-SD1-SD3 group showed decreasing trends. Compared with the C-NSD1-NSD3 group, three neutrophil-related differential proteins, four erythrocyte-related differential proteins and four enzyme-related differential proteins in the C-SD1-SD3 group showed upward trends.

Because the differences between the NS group and S group were clearest at Burn-day 1, we analyzed the differences in plasma proteins between groups on the first day after burn injuries. Differential proteins are shown in Figure 5g. GO enrichment analysis focused on four types of functions: cellular oxidant detoxification, hydrogen oxidant catabolic processes, cellular response to toxic substances and catabolic processes (Figure 5h).

### Validation study by PRM

**Figure 6:**
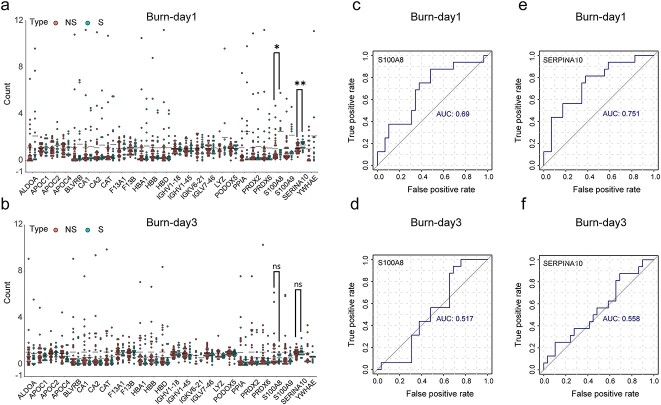
Proteomic revalidation of expanded samples by PRM assay. (**a**, **b**) PRM method was used to quantify 26 proteins of 45 patients on Burn-day 1 and Burn-day 3. (**c**, **d**) ROC curves of S100A8 protein on Burn-day 1 and Burn-day 3. (**e**, **f**) ROC curves of SERPINA10 protein on Burn-day 1 and Burn-day 3. ^*^p < 0.05, ^**^p < 0.01. *NS* burn group without sepsis, *S* burn group with sepsis, *AUC* area under the curve, *PRM* parallel reaction monitoring

From the DIA sequencing results, we found the differential proteins in NS and S groups on the first day of burn injuries. The specific information of burn patients is shown in Table 3. In addition, PCA results were used to identify the key time points (Burn-day 1 and Burn-day 3) to distinguish NS patients from S patients. Therefore, we further verified 26 differential proteins in 45 severe burn patients (NS group: *n* = 29, S group, *n* = 16) by PRM assay, to find a good early warning indicator of burn-related sepsis. The comparison of 26 differential proteins between DIA and PRM is shown in Table S3, see online supplementary material. The results showed that S100A8 and SERPINA10 proteins were statistically different between the NS group and the S group on the first day after burn injuries, but we did not find the same results on the third day after burn injuries (Figure 6a, b). In addition, ROC curve analysis showed that the area under the curve (AUC) of S100A8 and SERPINA10 proteins were 0.69 and 0.751 respectively on Burn-day 1 (Figure 6c, e), while the AUC of S100A8 and SERPINA10 proteins were 0.517 and 0.558, respectively, on Burn-day 3 (Figure 6d, f).

**Table 3:** Characteristics of burn patients grouped by sepsis according to PRM analysis. Data presented as median (age range); number (percentage)

**Characteristics**	**NS group (D1,3)**	**S group (D1,3)**
	*n* = 29	*n* = 16
Age, years	49 (18-83)	44.5 (28–83)
%TBSA	10–80	36–98
Degree of burn	II–III	II–III
Sex, male (%)	19 (65.52)	12 (75)
Inhalation injury (yes:no)	14:15	8:8

TBSA  total body surface area, *PRM* parallel reactionmonitoring

### Primary characterization of peripheral blood neutrophils in early severe burn patients

**Figure 7:**
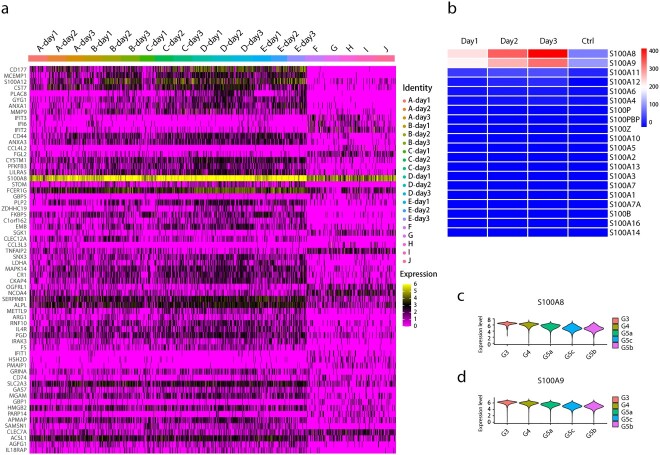
scRNA-seq analyses of healthy controls and severe burn injuries. (**a**) Heatmap of differential genes in subsets of neutrophils in healthy controls and severely burn injuries (days 1–3). Yellow represents high expression level, purple represents low expression level and black represents moderate expression level. (**b**) Heatmap of s100-related gene expression of neutrophils in healthy controls and severely burn injuries (days 1–3). (**c**, **d**) Violin plot of the genes S100a8 and S100a9 in different subsets of neutrophils

Previous studies focused on the relevant genes and functions of neutrophils. To further investigate changes in important cells in the early stages of severe burn injuries, we performed scRNA-seq on circulating neutrophils from healthy and diseased groups. The sequencing samples were collected from the peripheral blood neutrophils of five healthy controls and five patients at Burn-day 1, 2 and 3. The details of the samples have been described previously [36]. The transcriptomic changes in neutrophils after severe burn injuries, as compared with those in healthy controls, were dramatic, and the most pronounced change was observed for the S100A8 protein (Figure 7a).

In addition, other proteins of the S100 family were analyzed with a heatmap. The most pronounced differences were for the S100A8 and S100A9 proteins (Figure 7b). The calcium-binding proteins S100A8 and S100A9 belong to the S100 protein family and usually exist as S100A8/A9 heterodimers. In addition, our previous study has shown that many neutrophils enter the peripheral blood after burn injuries. Neutrophils in the peripheral blood were divided into five subgroups: mature neutrophils (G5a–c) and relatively immature neutrophils (G3 and G4). Compared with G5a–c, the G3 and G4 subgroups have more prominent activation and degranulation functions, which appear to aggravate burn severity [36]. In this study, a violin plot indicated that the S100A8 and S100A9 genes were also significantly upregulated in subgroups G3 and G4, and the effect was more significant for S100A8 (Figure 7c, d).

### Therapeutic effect of S100A8 inhibitor on severely burned mice

To explore the physiological changes in various organs in the early stages after severe burn injuries, we collected lung, spleen, liver and kidney tissues from mice at Burn-8 h (8 h after burn injury) and Burn-day 1 for H&E staining. The most significant change was observed in the lungs. Alveolar wall thickening was observed at Burn-8 h, and was more pronounced at Burn-day 1 and was accompanied by partial alveolar collapse (Figure S1a, see online supplementary material). For the spleen, progressive disorder of the white medullary structure and enlargement of the spleen was observed at Burn-8 h and Burn-Day1 (Figure S1b). In the liver, no significant changes were observed in parenchymal cells at Burn-8 h, while enlargement and hyperchromatism of liver parenchymal cells were observed at Burn-day 1 (Figure S1c). Compared with normal mouse kidneys, no clear pathological changes were observed in kidneys at Burn-8 h and Burn-day 1 (Figure S1d).

**Figure 8:**
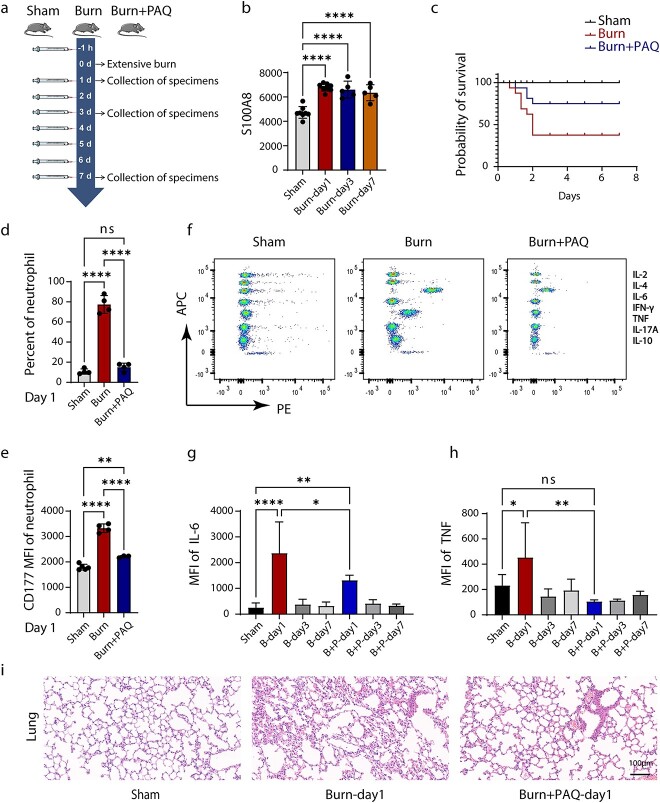
Therapeutic effect of S100A8 inhibitor on severely burned mice. (**a**) Schematic diagram of experimental design and sample collection strategy. Mice were scalded on 30% of their body area and then injected with PAQ at different time points. Blood and organ samples were collected at various points. (**b**) Expression of S100A8 in peripheral blood plasma of different groups of mice. (**c**) Survival rate change in severe burn mouse model. C57BL6/J mice with severe burn injury were randomized in three groups: sham group (Sham, *n* = 12), severe burn injury group (Burn, *n* = 16) and severe burn injury with PAQ group (Burn+PAQ, *n* = 16). (**d**) The proportion of peripheral blood neutrophils in each group on the first day of surgery. (**e**) CD177 expression of neutrophils in each group on the first day of operation. (**f**) Flow cytometry of the expression of seven inflammatory factors in peripheral blood plasma (IL-2, IL-4, IL-6, IFN-γ, TNF, IL-17A and IL-10). (**g**, **h**) The mean fluorescence intensity of inflammatory factors IL-6 and TNF in each group. (**i**) Histopathology of mouse lung tissue in each group (Hematoxylin–eosin staining, scale bar: = 100 μm). Data represent means ± SD (*n* ≥ 3) of two independent experiments. *p < 0.05, **p < 0.01, ****p < 0.0001, ns = not statistically significant compared with the control group. *PAQ* paquinimod, *MFI* mean fluorescence intensity, *IL* Iterleukin, *IFN-γ* interferon-γ, *TNF* tumor necrosis factor

S100A8 was highly expressed in the peripheral blood of mice and humans after severe burn injuries (Figure 8b). Previous studies have confirmed the early warning function of S100A8 at both gene and protein levels. To explore the therapeutic effects of S100A8, we used the S100A8 inhibitor PAQ in severely burned mice and assessed their survival rates. PAQ significantly increased the survival rate in mice with severe burn injuries (Figure 8c). To further explore the pathway involving PAQ, we detected the proportion of neutrophils in the peripheral blood in mice, the expression of CD177 on neutrophils and the expression of various inflammatory factors (IL-2, IL-4, IL-6, IFN-γ, TNF, IL-17A and IL-10) in the peripheral blood plasma. On the first day after burn injuries, PAQ intervention effectively inhibited the proportion of peripheral neutrophils (Figure 8d) and decreased CD177 expression on the membranes of peripheral neutrophils (Figure 8e). In addition, on the first day after burn injuries, PAQ effectively inhibited the expression of the inflammatory cytokines IL-6 and TNF in the peripheral blood, but not that of IL-2, IL-4, IFN-γ, IL-17A and IL-10 (Figure 8f–h and Figure S2 e–i, see online supplementary material). However, the same results were not observed in burned mice at Burn-Day3 and Burn-Day7 (Figure S2a–d). Meanwhile, PAQ treatment partially ameliorated lung injuries at Burn-Day1, including decreasing lung tissue edema and collapse (Figure 8i).

## Discussion

Sepsis after burn injuries is the most common complication in severe burn patients and is a major cause of multiple organ dysfunction syndrome [37,38]. Burn-associated sepsis tends to be aggressive, rapid and fatal. Therefore, early and accurate warning indicators, and timely diagnosis and effective prevention, of burn-associated sepsis are key to increasing the success rate of burn treatment. In recent years, the rapid development of multi-level omics biological technologies and computer analytic tools has provided a strong technical foundation for the early warning identification and treatment of diseases. In this multi-center, prospective multi-omics study, we performed whole blood WES, plasma miRNA sequencing, plasma proteomics DIA sequencing, plasma protein PRM sequencing and peripheral blood neutrophil scRNA-seq. Our aim was to perform a multi-omics search for biomarkers for early warning and treatment of burn-associated sepsis, to decrease the incidence of severe burn-associated sepsis and increase patient survival rates.

In the past 10 years, studies on gene polymorphisms in diseases have significantly increased, and WES is often used for genomic detection because of its high sensitivity and accuracy in detecting mutations. In this study, to identify susceptibility genes in severe burn patients complicated with sepsis, we performed WES on samples from 40 patients with severe burn injuries. Through mutation analysis, we found seven genes with significant differences (DNAH11, LAMA2, ABCA2, ZFAND4, CEP290, MUC20 and ENTPD1). The DNAH11, ZFAND4, LAMA2 and ENTPD1 genes have been shown to have protective effects against sepsis. ENTPD1 alleviates sepsis-associated liver injury by eliminating Extracellular adenosine triphosphate (eATP) [39]. Enhancement in ENTPD1 decreases lipopolysaccharide (LPS)-induced renal tubular epithelial cell injury, increases cell viability or apoptosis and inhibits NOD-like receptor thermal protein domain associated protein 3 (NLRP3) inflammasome activation [40]. Cohen *et al*. have shown that macrophage-specific ENTPD1 plays an important role in preventing LPS-induced fatal hyperinflammation *in vivo* [41]. In endothelial cells, overexpression of ENTPD1 effectively reverses LPS-induced ATP production and significantly inhibits IL-1α secretion [42]. Those four studies have emphasized the protective role of ENTPD1 in sepsis, thus providing strong evidence that ENTPD1 is a susceptibility gene for severe burn patients complicated with sepsis. In addition, ZFAND4 gene polymorphisms are strongly associated with acute lung injury in mice [43]. Using liquid chromatography-electrosprayionization-mass spectrometry/mass spectrometry (LC-ESI-MS/MS), Thavarajah *et al*. have found that the plasma peptide DNAH11 is strongly associated with sepsis [44]. A study by Li *et al*. has identified LAMA2 as a target for the treatment of toxic shock syndrome [45]. In the present study, mutations in ENTPD1, ZFAND4, DNAH11 and LAMA2 increased the risk of comorbid sepsis in severely burned patients. All the above studies provide strong evidence that these four genes are susceptibility genes for sepsis in severe burn patients. We did not find relevant literature reports regarding the roles of the other three genes (ABCA2, CEP290 and MUC20) in sepsis; therefore, these genes may provide new intervention targets for severe burn injuries complicated with sepsis.

At one time, miRNAs were considered genetic waste products because they are not translated into peptide chains/proteins. However, recent studies have suggested that they play important roles in regulating protein expression, and some studies have shown that miRNAs can be used as detection markers for various diseases. In this study, to search for warning markers for severe burn patients complicated with sepsis, we performed miRNA sequencing on the peripheral blood plasma of 40 severe burn patients and identified four differentially upregulated miRNAs (hsa-miR-16-5p, hsa-miR-185-5p, hsa-miR-451a and hsa-miR-423-5p). hsa-miR-16-5p has been demonstrated to be associated with sepsis [46]. In addition, the upregulation of circulating hsa-miR-16-5p has been found to increase the risk of chronic lymphocytic leukemia and gout [47,48]. The other three miRNAs have not been reported to be associated with sepsis and therefore may serve as novel early warning markers of burn-associated sepsis in the future.

Severe burn injury is a complex physiological process whose pathophysiological changes have not been fully clarified [49]. To further understand the key features of the onset and progression of the disease, we performed DIA detection on peripheral blood plasma from healthy controls and severe burn patients at different time points. The characteristics and commonalities of proteomics at different time points in severe burn patients were assessed. The difference in PCA at different time points after severe burn injuries reflects disease progression. The functional differences on Burn-day 1 were most significant and included mainly neutrophil degranulation, complement activation and red blood cell damage, as determined by the particular characteristics of severe burn injuries. After severe burn injury, the body suddenly changes from a relatively healthy state to a serious disease state, thereby explaining why the changes in differentially present proteins were most significant on the first day after injuries. In addition, we observed commonalities between time points after severe burn injuries. The discovery of 49 common differential proteins suggested that neutrophils, platelets and red blood cells all play crucial roles in the development of the disease. Consequently, subsequent interventions for severe burn injuries may focus on the relevant differential proteins and functional changes in these three cell types.

Proteins are the main executors of biological functions. Therefore, we searched for early warning markers of sepsis in severe burn patients at the protein level. By analyzing the main proteins in the NS group and S group at different time points, we found that the first day after severe burn injuries is the key time point to distinguish whether severe burn patients will develop sepsis. Further analysis of the NS group and the S group on Burn-day 1 showed that the functional enrichment of differential proteins in the peripheral blood plasma of the S group, compared with the NS group, reflected a more prominent cytotoxic reaction, which appeared to be one reason for sepsis in severe burn patients. Because targeted PRM detection has higher accuracy than non-targeted DIA sequencing, we conducted PRM revalidation for the 26 differential proteins screened by DIA sequencing. The validation results suggested that S100A8 and SERPINA10 may serve as biomarkers for severe burn patients with sepsis.

After severe burn injuries, the body enters a pathological condition of excessive inflammation, hypermetabolism and acute immune response [50]. Many immune cells enter the peripheral blood, and the most rapid response is exhibited by neutrophils. In addition, our previous studies have revealed that neutrophils play a crucial role in vascular leakage in severe burn injuries [51]. Therefore, to comprehensively describe the transcriptional landscape of neutrophil heterogeneity and functional multiplicity in the early stages of severe burn injuries, we performed scRNA-seq on neutrophils. Unexpectedly, scRNA-seq revealed extensive changes in the genomics of neutrophils after severe burn injuries, primarily in S100A8. In addition, S100A8 was more significantly expressed in the relatively immature neutrophil subpopulation. S100A8/9 can also be used as a prognostic diagnostic and therapeutic target for myocardial infarction [52]. These findings appear to suggest that S100A8 is not only a warning indicator of severe burn sepsis but also may have therapeutic effects in patients with severe burn injuries. To further verify the therapeutic effect of S100A8, we treated severely burned mice with intraperitoneal injection of the S100A8 inhibitor PAQ. S100A8 was also a therapeutic target, and its therapeutic effect was reflected by its effectively increasing the survival rate among severely burned mice, inhibiting the mobilization and activation of peripheral blood neutrophils at Burn-day1, ameliorating lung injury and inhibiting the release of the inflammatory factors IL-6 and TNF.

## Conclusions

In conclusion, we conducted a multi-center, prospective multi-omics study of severe burn patients to screen for prevention and treatment biomarkers for severe burn patients complicated by sepsis. At the genetic level, seven susceptibility genes for sepsis in severe burn patients were found: DNAH11, LAMA2, ABCA2, ZFAND4, CEP290, MUC20 and ENTPD1. In addition, we identified four miRNAs (hsa-miR-16-5p, hsa-miR-185-5p, hsa-miR-451a and hsa-miR-423-5p) that may serve as warning indicators of burn-associated sepsis. At the protein level, the proteins S100A8 and SERPINA10 were found to be biomarkers for severe burn patients with sepsis, and the results of scRNA-seq also revealed the important role of neutrophils in the early stages of severe burn injuries. In addition, we confirmed the role of the neutrophil-associated protein S100A8 in the early warning and treatment of severe burn-associated sepsis. Our study provides good biomarkers for early warning of severe burn-associated sepsis and a good potential clinical therapeutic target for the early treatment of severe burn injuries.

## Abbreviations

AUC: Area under the curve; DIA: Data-independent acquisition; GO: Gene ontology; H&E: Hematoxylin–eosin; PAQ: Paquinimod; PCA: Principal component analysis; PRM: Parallel reaction monitoring; ROC: Receiver operating characteristic; scRNA-seq: Single-cell transcriptome sequencing; WES: Whole exome sequencing; miRNA: micro-Ribonucleic acid; SNV: Single nucleotide variants; INDEL: Insertion and deletion; BCA: Bicinchoninic acid; SDS-PAGE: Sodium dodecyl sulfate polyAcrylamide gel electrophoresis; LC-MS/MS: Liquid chromatography-mass spectrometry/mass spectrometry; DDA: Data dependent acquisition; DTT: Dithiothreitol; IAA: Iodoacetamide; LC-PRM/MS: Liquid chromatography-parallel reaction monitoring/mass spectrometry; AGC: Automatic gain control; HCD: Higher energy collision induced di ssociation; DMSO: Dimethyl sulfoxide; PBS: Phosphate buffered saline; ELISA: Enzyme-linkedimmunosorbent assay; IL-2: Interleukin-2; IFN-γ: Interferon-γ; TNF: Tumor necrosis factor; MAPK: Mitogenactivated protein kinases; NLRP3: NOD-like receptor thermal protein domain associated protein 3; eATP: Extracellular adenosine triphosphate; LPS: Lipopolysaccharide; LC-ESI-MS/MS: Liquid chromatographyelectrospray ionization-mass spectrometry/mass spectrometry. SDT is a lysate consisting of SDS and Tris. SDT has no corresponding acronym.

## Supplementary Material

Supplementary_files_tkac050

## Data Availability

All data used in this work is available upon reasonable request.
