# Supplementary material for: Prospective study and validation of early warning marker discovery based on integrating multi-omics analysis in severe burn patients with sepsis
Source: Burns Trauma. 2023 Jan 15;11:tkac050. doi: 10.1093/burnst/tkac050 (PMC9840905; doi:10.1093/burnst/tkac050)
Supplement: Supplementary_files_tkac050 [file supplementary_files_tkac050.docx]

### **Supplementary files**

**Title：****Prospective study of early warning markers based on integrating multiomics analysis in severe burn patients with** **sepsis**

**Authors:** Jiamin Huang, Yi Chen, Zaiwen Guo, Yanzhen Yu , Yi Zhang, Pingsong Li, Lei Shi, Guozhong Lv, Bingwei Sun

**Correspondence to:** Professor Bingwei Sun, MD, PhD, Department of Burns and Plastic Surgery, Affiliated Suzhou Hospital of Nanjing Medical University, Suzhou 215002, Jiangsu Province, China

**Telephone:** +86 512 6236 3018 **Fax:** +86 512 6236 3018

**Email:** [sunbinwe@hotmail.com](mailto:sunbinwe@hotmail.com)


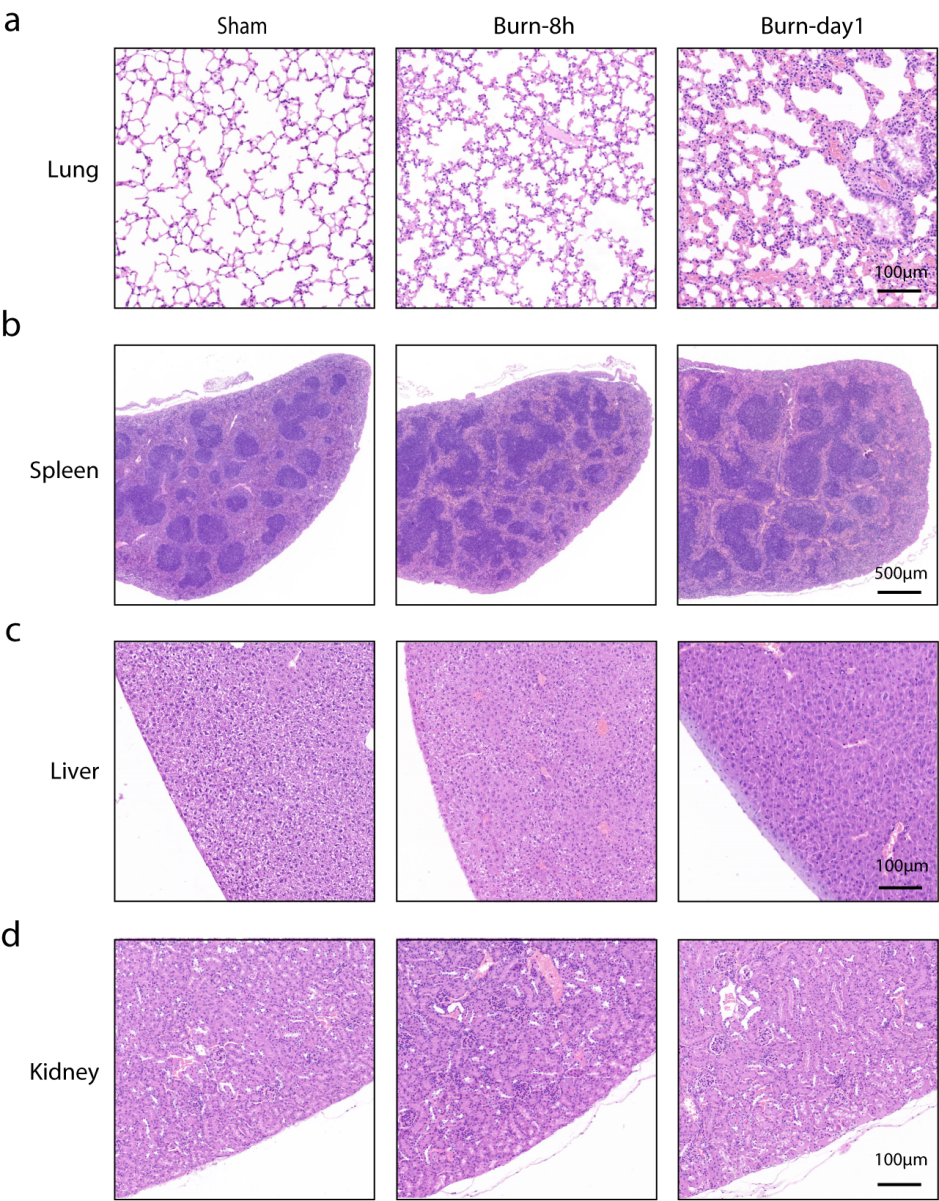


**Supplementary files Fig. 1** **Histopathological results of various organs in mice. a**,**c**,**d**, Histopathology of mouse lung, liver and kidney tissue in each group (HE]staining, bar=100 μm).**b**, Histopathology mouse spleen tissue in each group (HE staining, bar=500 μm).


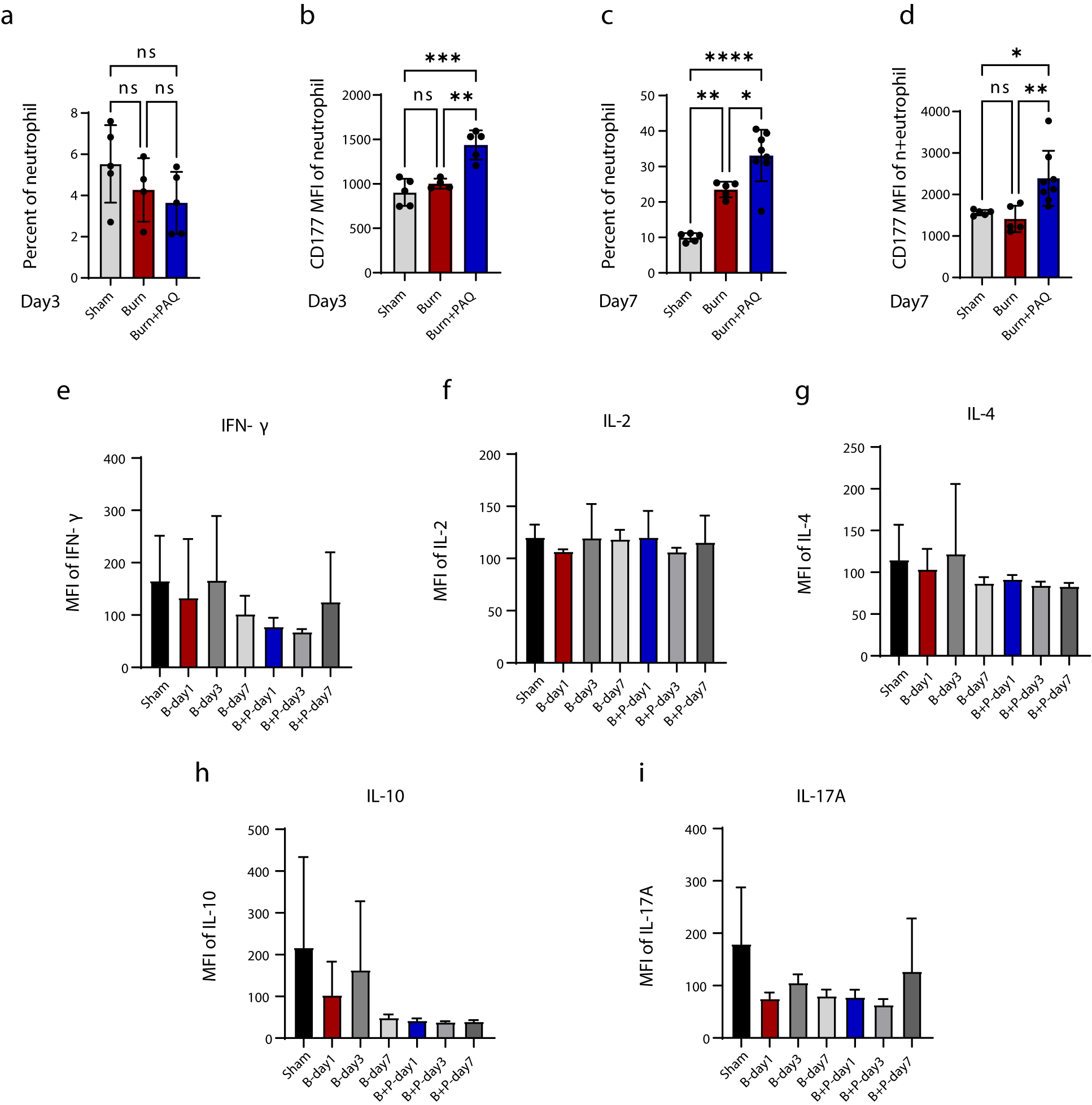


**Supplementary files Fig. 2 Effects of s100A8 inhibitor on severely burned mice at day 3 and day 7. a**,**c,** The proportion of peripheral blood neutrophils in each group on the third and seventh day of surgery. **b**,**d,** CD177 expression of neutrophils in each group on the third and seventh day of operation. **e-i**, The mean fluorescence intensity of inflammatory factors IL-2, IL-4, IFN-γ, IL-17A and IL-10 in each group. Data represent means ± s.d. (n≥3) of two independent experiments. *p < 0.05, p** < 0.01, ***p < 0.001,****p < 0.0001. ns=not statistically significant compared with the control group; PAQ=paquinimod; MFI=mean fluorescence intensity.

**Supplementary files Table 1. Differential proteins for trend analysis**

| ProteinAccessions | Genes | [Pattern](javascript:;)  (C-NSD1-NSD3) | [Pattern](javascript:;)  (C-SD1-SD3) | Trend |
| --- | --- | --- | --- | --- |
| A0A075B6I9 | IGLV7-46 | NA | 1 | Down |
| A0A0B4J1V0 | IGHV3-15 | NA | 1 | Down |
| A0A0C4DH24 | IGKV6-21 | NA | 0 | Down |
| A0A0C4DH39 | IGHV1-58 | NA | 3 | Down |
| A0A0G2JS06 | IGLV5-39 | NA | 3 | Down |
| A2NJV5 | IGKV2-29 | NA | 1 | Down |
| P00441 | SOD1 | NA | 6 | Up |
| P00734 | F2 | NA | 1 | Down |
| P00915 | CA1 | 5 | 6 | Up |
| P00918 | CA2 | 5 | 6 | Up |
| P01700 | IGLV1-47 | NA | 1 | Down |
| P01721 | IGLV6-57 | NA | 0 | Down |
| P01833 | PIGR | NA | 6 | Up |
| P02671 | FGA | NA | 7 | Up |
| P02743 | APCS | NA | 4 | Up |
| P02768 | ALB | NA | 1 | Down |
| P02792 | FTL | NA | 4 | Up |
| P04003 | C4BPA | NA | 7 | Up |
| P04040 | CAT | 5 | 6 | Up |
| P04114 | APOB | NA | 7 | Up |
| P04430 | IGKV1-16 | NA | 4 | Up |
| P06702 | S100A9 | NA | 6 | Up |
| P06727 | APOA4 | NA | 3 | Down |
| P07384 | CAPN1 | NA | 6 | Up |
| P0DOX5 | NA | NA | 1 | Down |
| P0DOX8 | NA | NA | 1 | Down |
| P10124 | SRGN | 5 | 6 | Up |
| P11142 | HSPA8 | NA | 6 | Up |
| P14618 | PKM | NA | 6 | Up |
| P14780 | MMP9 | 5 | 6 | Up |
| P19827 | ITIH1 | NA | 1 | Down |
| P26022 | PTX3 | 5 | 4 | Up |
| P26038 | MSN | 5 | 6 | Up |
| P30041 | PRDX6 | 3 | 6 | Up |
| P30046 | DDT | NA | 6 | Up |
| P32119 | PRDX2 | 5 | 6 | Up |
| P35542 | SAA4 | NA | 4 | Up |
| P37837 | TALDO1 | 5 | 6 | Up |
| P55072 | VCP | NA | 6 | Up |
| P62258 | YWHAE | NA | 6 | Up |
| P69891 | HBG1 | 3 | 6 | Up |
| P69892 | HBG2 | 5 | 6 | Up |
| Q13093 | PLA2G7 | NA | 6 | Up |
| Q66K66 | TMEM198 | NA | 0 | Down |
| Q86U17 | SERPINA11 | 2 | 3 | Down |
| Q86YZ3 | HRNR | 5 | 6 | Up |
| Q8NHQ9 | DDX55 | NA | 0 | Down |
| Q9HCS7 | XAB2 | NA | 1 | Down |

C=control; NS=burn group without sepsis; S=burn group with sepsis; C-NSD1-NSD3=C group to NS group-Day1 to NS group-Day3; C-SD1-SD3=C group to S group-Day1 to S group-Day3; NA=not applicable.

**Supplementary files Table 2. Functional annotation of differential proteins for trend analysis**

| Genes | Trend | [Function](javascript:;) [annotation](javascript:;) | [References](javascript:;) |
| --- | --- | --- | --- |
| IGLV7-46  IGHV3-15  IGKV6-21  IGHV1-58  IGLV5-39  IGKV2-29  IGLV1-47  IGLV6-57 | Down | Immunoglobulins, also known as antibodies, are membrane-bound or secreted glycoproteins produced by B lymphocytes. They are primarily involved in the immune response. | 16-20 |
| SRGN  MMP9  S100A9 | Up | They are neutrophil-associated proteins that are associated with neutrophil mobilization, infiltration, activation, and degranulation. | 21-25 |
| HBG1  HBG2  CA1  CA2 | Up | They are red blood cell-associated proteins that are involved in the heme/hemoglobin metabolism pathway, blood oxygen transport and carbon dioxide metabolism. | 26-29 |
| SOD1  CAT  PRDX6  TALDO1 | Up | They are all enzymes, and their main role is to destroy free superoxide free radicals in the body and reduce the toxic effects of hydrogen peroxide. | 30-33 |

**Supplementary files Table 3. Comparison of targeted protein expression according to DIA and PRM methods**

| Protein | Gene | DIA-S vs NS FC | DIA-S vs NS log2FC | DIA  P. Value | PRM-S vs NS FC | PRM-S vs NS  log2FC | PRM  P. Value | Consistency of DIA and PRM |
| --- | --- | --- | --- | --- | --- | --- | --- | --- |
| P04075 | ALDOA | 4.0996 | 2.0355 | 0.0014 | 1.9369 | 1.0175 | 0.2247 | Yes |
| P02042 | HBD | 3.5774 | 1.8389 | 0.0021 | 0.9976 | -0.0027 | 0.9533 | Yes |
| P69905 | HBA1 | 2.9273 | 1.5496 | 0.0017 | 1.1347 | 0.1477 | 0.6814 | Yes |
| P32119 | PRDX2 | 4.1716 | 2.0606 | 0.0017 | 1.1108 | 0.1208 | 0.8604 | Yes |
| P04040 | CAT | 3.2914 | 1.7187 | 0.0037 | 1.2508 | 0.2743 | 0.7338 | Yes |
| P30043 | BLVRB | 6.4811 | 2.6962 | 0.0375 | 1.2779 | 0.3046 | 0.8055 | Yes |
| P00915 | CA1 | 3.5400 | 1.8238 | 0.0042 | 1.0814 | 0.0890 | 0.6473 | Yes |
| P62937 | PPIA | 4.7550 | 2.2495 | 0.0107 | 2.2829 | 1.3962 | 0.1642 | Yes |
| P30041 | PRDX6 | 9.7310 | 3.2826 | 0.0192 | 0.9880 | -0.0131 | 0.8604 | Yes |
| P00918 | CA2 | 3.1103 | 1.6371 | 0.0111 | 1.2112 | 0.2303 | 0.6305 | Yes |
| P05109 | S100A8 | 3.5503 | 1.8279 | 0.0074 | 2.4942 | 1.4990 | 0.0393 | Yes |
| P06702 | S100A9 | 3.9845 | 1.9944 | 0.0039 | 2.1148 | 1.1424 | 0.1101 | Yes |
| P68871 | HBB | 2.0809 | 1.0572 | 0.0145 | 0.9918 | -0.0090 | 1.0000 | Yes |
| P62258 | YWHAE | 2.6271 | 1.3935 | 0.0000 | 2.1094 | 1.1094 | 0.3842 | Yes |
| P55056 | APOC4 | 1.8486 | 0.8864 | 0.0423 | 1.0917 | 0.0965 | 0.8055 | Yes |
| P61626 | LYZ | 1.5817 | 0.6615 | 0.1337 | 1.9755 | 0.9755 | 0.3528 | Yes |
| P02655 | APOC2 | 1.4452 | 0.5313 | 0.1495 | 1.2307 | 0.2384 | 0.4738 | Yes |
| P0DOX5 | - | 0.6968 | -0.5211 | 0.1479 | 1.0300 | 0.0300 | 0.7338 | No |
| A0A0C4DH31 | IGHV1-18 | 0.8627 | -0.2130 | 0.4916 | 0.9537 | -0.0474 | 0.9160 | Yes |
| Q9UK55 | SERPINA10 | 0.6574 | -0.6051 | 0.0101 | 1.3055 | 0.3116 | 0.0062 | No |
| P05160 | F13B | 0.6238 | -0.6809 | 0.0635 | 1.1072 | 0.1035 | 0.3780 | No |
| P00488 | F13A1 | 0.5033 | -0.9904 | 0.0189 | 1.0215 | 0.0221 | 0.7914 | No |
| A0A0A0MS14 | IGHV1-45 | 0.5181 | -0.9487 | 0.0074 | 1.0186 | 0.0185 | 0.5339 | No |
| A0A075B6I9 | IGLV7-46 | 0.5671 | -0.8184 | 0.0349 | 1.0756 | 0.0767 | 0.8789 | No |
| P02654 | APOC1 | 2.2281 | 1.1558 | 0.2695 | 1.1238 | 0.1242 | 0.6987 | Yes |
| A0A0C4DH24 | IGKV6-21 | 0.7693 | -0.3784 | 0.8421 | 1.0739 | 0.0749 | 0.4938 | No |

DIA=data-independent acquisition; PRM=parallel reaction monitoring; NS=burn group without sepsis; S=burn group with sepsis; FC=fold change.
